# Lipopolysaccharide Upregulated Intestinal Epithelial Cell Expression of Fn14 and Activation of Fn14 Signaling Amplify Intestinal TLR4-Mediated Inflammation

**DOI:** 10.3389/fcimb.2017.00315

**Published:** 2017-07-11

**Authors:** Xuefeng Qi, Lijuan Qin, Ruijing Du, Yungang Chen, Mingzhu Lei, Meiyu Deng, Jingyu Wang

**Affiliations:** College of Veterinary Medicine, Northwest A&F University Yangling, China

**Keywords:** small intestinal epithelial cells, TLR4, Fn14, TWAEK, TNF-α, lipopolysaccharide

## Abstract

TLR4 in intestinal epithelial cells has been shown both inflammatory and homeostatic roles following binding of its cognate ligand lipopolysaccharide (LPS). TWEAK-Fn14 axis plays an important role in pathologies caused by excessive or abnormal inflammatory responses. This study aimed to evaluate potential cross-talk between TLR4 and TWEAK/Fn14 system in porcine small intestinal epithelial cells. Our *in vivo* results showed that, compared with the age-matched normal control piglets, increased expression of Fn14 in epithelium and decreased TWEAK expression in lamina propria were detected in the small intestinal of piglets stimulated with LPS. Consistent with this finding, treatment with LPS increased the expression of Fn14 and TLR4 while decreased TWEAK expression in porcine small intestinal epithelial cell lines SIEC02. Interestingly, modulating Fn14 activation using agonistic anti-Fn14 decreased TLR4-mediated TNF-α production by SIEC02. In addition, pretreatment of LPS-stimulated SIEC02 with recombinant TWEAK protein suppresses the expression of Fn14 and TNF-α and inhibits the negative impact of LPS on the tight junctional protein occludin expression. In conclusion, this study demonstrates that the TWEAK-independent Fn14 activation augments TLR4-mediated inflammatory responses in the intestine of piglets. Furthermore, the TWEAK-dependent suppression of Fn14 signaling may play a role in intestinal homeostasis.

## Introduction

Intestinal epithelial cells play a critical role in the activation and regulation of intestinal inflammation. Toll-like receptors (TLRs) are critical components of the innate immune response that detect microorganisms through the recognition of pathogen-associated molecular patterns (PAMPs) (Magalhaes et al., 2007). Studies performed to characterize signal transduction pathways of TLRs in intestinal epithelial cells have shown both inflammatory and homeostatic roles for these receptors following of their cognate ligands binding (Kamdar et al., 2013). TLR4 expressed on intestinal epithelial cells have shown pro-inflammatory role by specifically recognizing lipopolysaccharide (LPS). The intracellular signaling of TLR4 has been mainly divided into TIRAP-MyD88 and TRIF-TRAM pathways (Akira and Takeda, 2004; Kawai and Akira, 2010). Recent study demonstrates that LPS-induced inflammation and mucosal damage is mediated by activation of the TLR4/FAK/MyD88 signal transduction axis (Guo et al., 2015).

TNF-like weak inducer of apoptosis (TWEAK) is a cytokine of the tumor necrosis factor (TNF) ligand superfamily. Accumulating evidence in humans and mice suggest that TWEAK might have an important role in cell proliferation, cell migration, inflammation and tissue homeostasis through its receptor fibroblast growth factor-inducible molecule 14 (Fn14) (Han et al., 2003; Tran et al., 2005, 2006; Brown et al., 2013). TWEAK is expressed broadly by many immune cell types, including monocytes/macrophages, dendritic cells, neutrophils, natural killer cells and T cells. On the other hand, Fn14 is widely expressed at relatively low levels in healthy tissues, but Fn14 upregulation has been detected following tissue injury and chronic inflammatory, which indicating the activation of the TWEAK/Fn14 pathway in the context of tissue injury and inflammation occurs through induction of Fn14 (Winkles, 2008; Burkly, 2014). Although the highly inducible nature of Fn14 and the propensity for Fn14 up-regulation in disease conditions is established, factors that could have dramatic effects on the stability of Fn14 or mechanisms underlying Fn14 down-regulation have not been fully investigated.

Upregulation of receptor expression may either increase the sensitivity to ligand or activate the ligand-independent signaling by the receptor (Han et al., 2003; Tran et al., 2005, 2006; Brown et al., 2013). Several *in vitro* studies have suggested that ectopically over expressed Fn14 can signal in a ligand-independent manner (Brown et al., 2003, 2013; Han et al., 2003). It has been reported that, in mouse tissues and peritoneal macrophages, the expression levels of TWEAK mRNA rapidly decreased in response to the treatment of LPS (Chicheportiche et al., 2000). In addition, previous *in vitro* studies indicated that Fn14 may plays a role in amplifying inflammation in the lower genital tract during infection (Han et al., 2010; Qi et al., 2016). These studies, however, do not fully elucidate the mechanisms underlying the TWEAK-independent Fn14 pathway in the pathophysiology of inflammatory diseases. In the current study, we sought to determine whether TWEAK-independent Fn14 pathway plays a role in the amplification of LPS-induced inflammation in porcine small intestinal epithelial cell lines SIEC02.

## Materials and methods

### Ethics statement and experimental animals

The animal experiments were carried out in strict accordance with guidelines established by the Ethics of Animal Experiments of Northwest A&F University, Yangling, China. All the protocols were approved by this committee (Permit Number: 2014BAD23B11).

Healthy unsuckled 1-day-old Landrace piglets were housed individually in separate pens and were weaned at 28 days of age. Weanling piglets had *ad libitum* access to feed and water.

### Experimental design and sample collection

Small intestine tissues were obtained from 1-day-old (neonatal, *n* = 3), 7-day-old (suckling, *n* = 3), and 28-day-old (weaned, *n* = 3) healthy piglets for target genes expression analysis. In addition, the protocol for LPS-induced inflammatory in piglets was performed as described previously (Jiang et al., 2009). Briefly, piglets at the age of 52 days were injected intraperitoneally with either LPS (*Escherichia coli* 055:B5, Sigma Chemical) at 200 μg/kg body weight or an equivalent amount of sterile saline. Piglets showed diarrhea on 3 day post injected with LPS. The control piglets did not manifest any signs of disease throughout the experiment.

Piglets were humanely killed by captive bolt and exsanguinated on the designated days and their small intestine tissues were dissected. A 10-cm small segment of small intestine was excised, sliced into smaller fractions, frozen immediately in liquid nitrogen, and then stored at −80°C until analysis of mRNA abundance and protein profiles. Tissues for immunofluorescence analysis were fixed in 10% buffered formalin (Sigma-Aldrich, Oakville, ON, Canada) for 48 h and then processed and embedded in paraffin.

### Cell culture and treatment

The porcine small intestine epithelial cell line SIEC02 (Wang et al., 2014) was kindly provided by Prof. YM Zhang (Northwest A&F University, Yangling, PR China) and cultured in complete culture solution (DMEM:F12 medium = 1:1, 10% FCS; Gibco BRL, Life Technologies, Grand Island, NY, USA). When the cells reached 80–90% confluent, they were exposed to LPS (Sigma-Aldrich) at the indicated concentrations. The negative control was incubated with DMEM:F12 medium and three parallel replicates were used for each group. To determine the role of TWEAK-independent and TWEAK-dependent Fn14 signaling in LPS-induced intestinal inflammation, 10^6^ porcine small intestinal epithelial cells were seeded into each well of a 24-well plate and stimulated with LPS for 24 h. Then, recombinant TWEAK (Abcam, Cambridge, MA, USA), Fn14 antibody (Abcam), or a TNF-α antibody (Abcam) was added at the indicated final concentrations and the cells were collected for analyses after 24 h incubation. Isotype control IgG was used as control in multiple blocking antibodies experiments. Small interfering RNA (siRNA)-mediated knockdown of Fn14 gene in SIEC02 was also used to determine the role of Fn14 in the present culture system.

### Immunofluorescent staining

Paraffin sections 5 μm in thickness were deparaffinized in xylene and rehydrated prior to analysis. The slides were washed, rehydrated, and nonspecific binding was blocked using 5% normal goat serum in wash buffer for 1 h. Intestinal epithelial cells grown on cover slips were fixed in 4% PFA (5 min at RT) and permeabilized in 0.1% Triton-X (10 min at RT). The cells were washed and incubated overnight with the following primary antibodies at 4°C: rabbit anti-TWEAK (1:100, Abcam); mouse anti-Fn14 (1:75, Abcam), or mouse anti-Occludin (1:200, Santa Cruz Biotechnologies, Santa Cruz, CA, USA). The sections were washed and incubated (2 h at RT) with a FITC-conjugated goat anti-rabbit secondary antibody (1:50, ThermoFisher). The sections were then incubated (2 h at RT) with Cy3-conjugated goat anti-mouse secondary antibody (1:75, ThermoFisher, Philadelphia, PA, USA), treated with 20 mM L-lysine (Sigma) to quench the autofluorescence. Sections were then mounted with mounting medium containing the DNA-intercalating dye DAPI (4, 6-diamidino-2-phenylindole, Invitrogen). Images were collected using an AxioCam equipped Zeiss M1 Imager with Axiovision software (Care Zeiss; Toronto, ON, Canada)

### Small interfering RNA transfection

Small interfering RNA (siRNA) oligonucleotides specific for sus scrofa Fn14 mRNA (GenBank Accession number: EU417836.1) were as follows: 5′-CGC AGG AGA GAG AAG TTT A-3′. SIEC02 monolayer were transfected with Fn14 siRNA and non-targeting control siRNA using lipofectamine 2000 (Invitrogen) in accordance with the manufacturer's protocol. Briefly, cells (5 × 10^5^ cells/well) seeded into a 6-well plate were transfected with 50 nM of control or Fn14 siRNA (RIBOBIO, GuangZhou, China) using 5 μl of lipofectamine 2,000 and 500 μl of Opti-MEMI (Invitrogen) for 6 h following the manufacturer's instruction (Invitrogen). Then, the medium of the cells was replaced with fresh medium. The cells were harvested at indicated time points post transfection and knockdown of Fn14 expression was determined by Western blot analysis.

### Quantitative real-time RT-PCR

Total RNA was extracted from intestine tissues and porcine small intestinal epithelial cells using TRIzol reagent (Invitrogen, Waltham, MA, USA) according to the manufacturer's instructions. RNA was then reversed using Superscript III (Invitrogen) and random primers (Invitrogen). Real-time quantitative PCR was carried out using a 7,300 Real-time PCR System (Applied Biosystems, Warrington, UK) and Power SYBR Green PCR Master Mix (Applied Biosystems). The sequences of the primers used were as follows:

*TWEAK*, 5′-CTGTCAGGTGCACTTTGATGAG-3′ (forward), 5′-AGCAAGTCCAGCTTCAGGTAGA-3′ (reverse); *Fn14*, 5′-GCGCTCTGAGCCTGACCTTC-3′ (forward), 5′-GGTGGTGAACTTCTCTCTCCTGC-3′ (reverse); *TLR4*, 5′-CCTATGACGCCTTTGTTATCTACT-3′ (forward) 5′-GATACACCATCGGCTCTGTATG-3′ (reverse); *18S*, 5′-TTCGGAACTGAGGCCATGAT-3′ (forward) 5′-CGAACCTCCGACTTTCGT-3′ (reverse). The PCR cycling conditions were 20 s at 95°C; followed by 40 cycles of 3 s at 95°C and 30 s at 60°C. Expression of 18S rRNA housekeeping gene was used to normalize cDNA levels for differences in total cDNA levels in the samples. Then, the Ct (*d*) was used to calculate the fold difference in copy number using the formula *f* = 2^(−*d*)^, where *f* = the fold difference in the expression of a specific gene and *d* = the difference in the Ct values between the compared sources of mRNA (corrected for differences in the 18S rRNA levels). We normalized each sample to normal intestinal specimen#1. Melt curves were performed to confirm the purity of the amplified products.

### Western blot analysis

Protein homogenates from the intestine tissues and intestinal epithelial cells were extracted as previously described (Lei et al., 2015). Briefly, the intestine tissue was lysed with lysis buffer containing 50 mM Tris-HCl (pH 7.4), 150 mM NaCl, and 1% Triton X-100, supplemented with protease inhibitor cocktail tablets (Roche). For the intestinal epithelial cells, the cells were lysed for 20 min on ice in ice-cold lysis buffer (Roche). The lysates were centrifuged at 12,000 × g for 20 min at 4°C to obtain a clear lysate. The protein content of each sample was determined using the BCA Protein Assay Kit (Thermo Scientific). Then, equal amounts of protein were separated on a 12% SDS-polyacrylamide gel and transferred to polyvinylidene difluoride membranes. Membranes were probed overnight at 4°C with following primary antibodies: rabbit anti-TWEAK (1:2,000, Abcam), mouse anti-Fn14 (1:3,000, Abcam), mouse anti-Occludin (1:200, Santa Cruz), mouse anti-TLR4 (1:1,000, Abcam), rabbit anti-MyD88 (1:1,000, Abcam), mouse anti-RelA (1:1,500, Cell Signaling, Beverly, MA, USA), or rabbit anti-TNF-α (1:1,500, Abcam). The bands were visualized using HRP-conjugated goat anti-rabbit IgG (1:20,000, Boster, Wuhan, China) or HRP-conjugated goat anti-mouse IgG (1:15,000, Boster) prior to the ECL protocol (Amersham Biosciences Piscataway, NJ, USA). As an internal standard, all membranes stripped with primary antibodies were reprobed with anti-β-actin antibody (Invitrogen). Changes in protein expression were determined after normalizing the band intensity of each lane to that of β-actin. Signal was visualized using the Konica SRX 101A developer (Konica Minolta Medical Imaging, Wayne, NJ, USA) and the Quantity One software (Bio-Rad, Mississauga, ON, Canada) was used to densitometrical analysis.

### Flow cytometric assay

The changes of Fn14 expression cells among SIEC02 over 24 h incubated with different conditioned mediums in comparison with the controlled cells were analyzed by flow cytometry. Briefly, cells (1 × 10^6^) were incubated with Phycoerythrin-conjugated anti-Fn14 antibody (BD Biosciences) as described by the manufacturer's instructions. Then, the cells were washed twice in cold phosphate buffered saline (PBS) and analyzed on a FACSCalibur (BD Biosciences, San Jose, CA).

### Trypan blue dye exclusion method

The number of viable cells after 24 h TWEAK treatment was determined by using trypan blue dye exclusion assay. Cells were diluted 1: 1 (volume: volume) using Trypan Blue (Corning, catalog number: 25-900-CI) per manufacturer's instructions, before counting with a Neubauer hemocytometer.

### Statistical analyses

All experiments shown were performed independently a minimum of 3 times and all values are expressed as mean ± standard error of mean (SEM). Significance was determined by a one-way ANOVA with a Dunnett-post test, or by the Student paired *t*-test. The *P* < 0.01 are indicated by two asterisks (^**^). The *P* < 0.05 are indicated by one asterisks (^*^).

## Results

### Expression of TWEAK, Fn14, and TLR4 in the small intestine tissue of piglets

**Figure 1:**
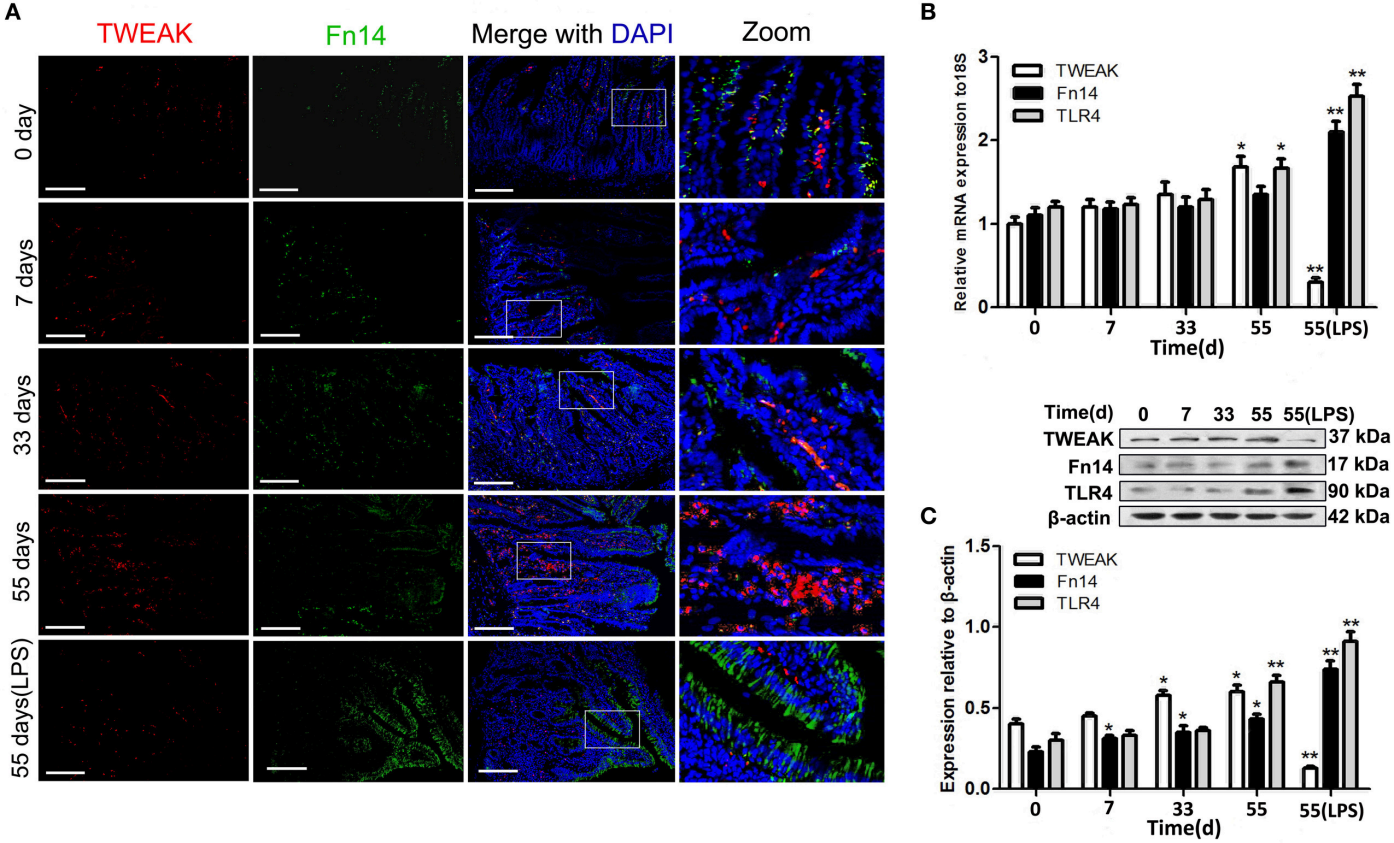
Location and expression of the tumor necrosis factor-like weak inducer of apoptosis (TWEAK) and fibroblast growth factor-inducible molecular (Fn14) in the small intestine of piglets. Representative fluorescence photomicrographs shows the co-localization of TWEAK (red) and Fn14 (green) in the small intestine from normal piglets on the indicated days and lipopolysaccharide (LPS)-treated piglets **(A)**. Nuclei were labeled with DAPI. Scale bars = 200 μm. The expression of TWEAK and its receptor Fn14 and TLR4 in the small intestine of piglets at the indicated days was analyzed by quantitative real-time RT-PCR **(B)** and Western blot analysis **(C)**. Representative blots are shown in the upper panel, and the histograms in the under panel show the results of densitometric analyses of TWEAK, Fn14 or TLR4 expression (c, normalized to β-actin to correct for protein loading). The data represent the mean ± SEM (*n* = 3). ^*^P < 0.05, ^**^P < 0.01, compared with data on 0-day-old.

Age-specific differences in the distribution and expression patterns of the TWEAK protein and Fn14 were determined in small intestine tissue from piglets. Immunofluorescence detection showed that slight labeling of TWEAK was mainly observed in the central lacteals in intestinal villus of the healthy piglets on 0-, 7-, and 33-day-old. Interestingly, immunostaining of TWEAK was visibly stronger in lamina propria of 55-day-old piglet small intestine (Figure 1A). For Fn14, weak staining was detected in the small intestine epithelium of healthy piglets on 0, 7, 33, and 55 days of old. In addition, small intestine tissue from LPS stimulated 55-day-old piglets was assessed for TWEAK and Fn14 expression. Conversely, very weak TWEAK staining in the central lacteals and strong Fn14 staining in the intestinal epithelial cells was observed in the LPS stimulated piglets compared with the age matched untreated control (Figure 1A).

Quantitative differences in TWEAK and Fn14, as well as TLR4 expression were assessed by quantitative real-time PCR and Western blot analysis. Both the mRNA and the protein expression levels of TWEAK and Fn14 undergo age-dependent up regulation in the healthy small intestine of piglets and showed similar trends as the histological assessments (Figures 1B,C). However, treatment of 55-day-old piglets with LPS up regulated Fn14 while down regulated TWEAK expression in the small intestine at both the mRNA and the protein levels. Similar expression profiles for TLR4 and Fn14 were observed from both untreated control and LPS stimulated small intestine (Figures 1B,C).

### LPS upregulates expression of Fn14 and TLR4 in small intestinal epithelial cells in a dose-dependent manner

**Figure 2:**
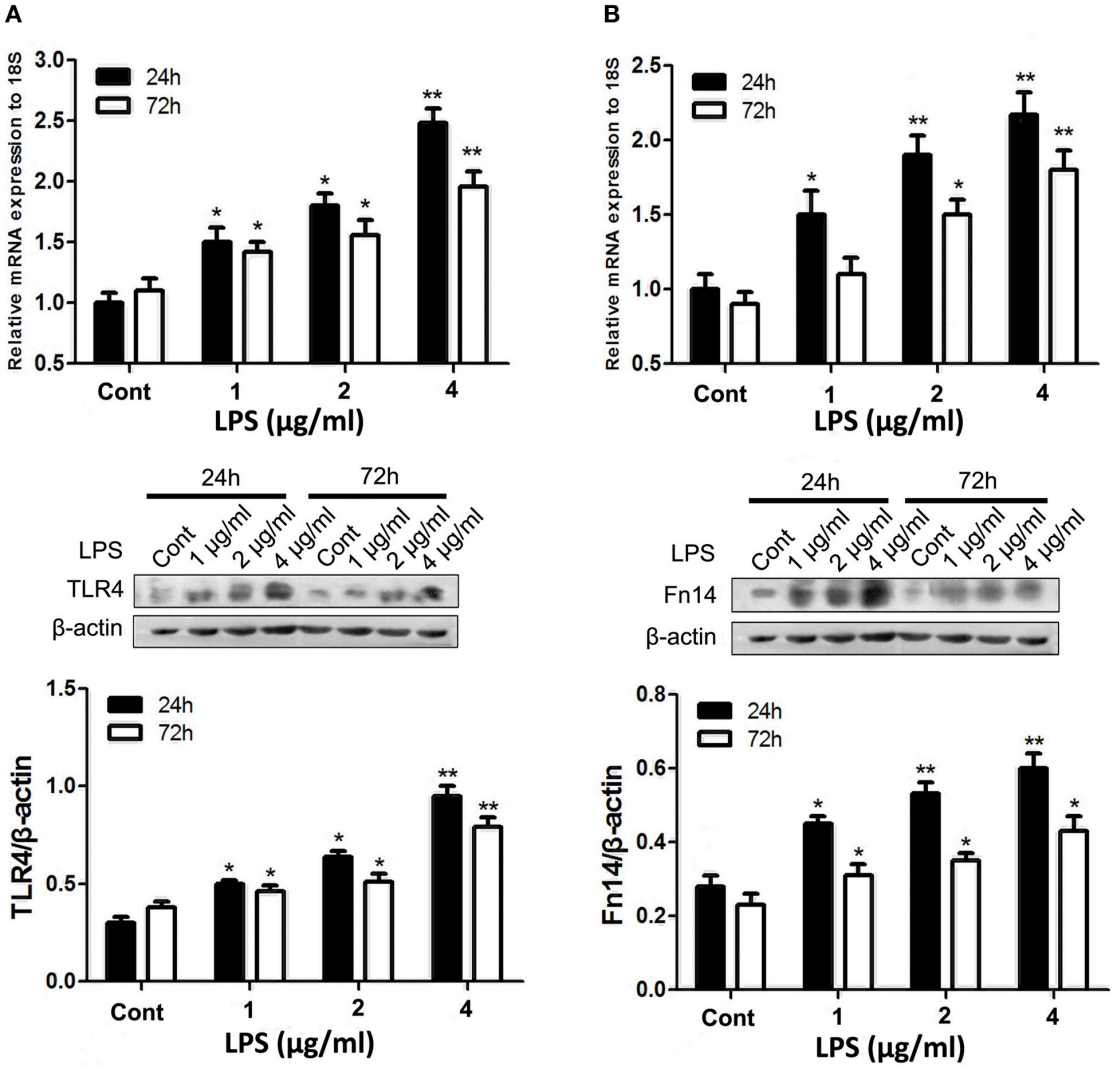
The expression of TLR4 and fibroblast growth factor-inducible molecule (Fn14) by porcine small intestinal epithelial cell lines (SIEC02) in response to LPS stimulation. The expression of TLR4 **(A)** and Fn14 **(B)** in the SIEC02 in response to the LPS treatment was analyzed by quantitative real-time RT-PCR and Western blot analysis. Representative blots are shown in the upper panel and the histograms in the under panel show the results of densitometric analysis of TLR4 **(A)** and Fn14 **(B)** expression (normalized to β-actin to correct for protein loading). The data represent the mean ± SEM (*n* = 3). ^*^P < 0.05, ^**^P < 0.01, compared with data on 0-day-old.

Given that strong Fn14 was mainly expressed in the intestinal epithelial cells and TLR4 is well characterized as key innate immune sensors in the porcine small intestine (Vaure and Liu, 2014), we directly determine the cross-talk between Fn14 and TLR4 expression in intestinal epithelial cells. We first sought to determine whether LPS stimulation upregulates both Fn14 and TLR4 expression in intestinal epithelial cells. The effects of LPS stimulation on Fn14 and TLR4 expression levels were performed by using SIEC02 that retained the morphological and functional characteristics of primary swine intestinal epithelial cells (Wang et al., 2014). As shown in Figure 2, upregulation of both TLR4 and Fn14 in SIEC02 were detected following stimulation with LPS. We confirmed that treatment of SIEC02 with increasing concentrations of LPS for either 24 or 72 h, both resulted in a dose-dependent upregulation of TLR4 at both the mRNA and the protein levels (Figure 2A). However, TLR4 levels in LPS stimulation of SIEC02 for 24 h were higher compared that of 72 h treatment group. Analysis of Fn14 levels after time-course treatment of SIEC02 with different concentrations of LPS stimulation revealed similar expression patterns with that of TLR4 expression at both mRNA and protein levels (Figure 2B).

### LPS induces upregulation of Fn14 in SIEC02 in a TNF-α-dependent manner

**Figure 3:**
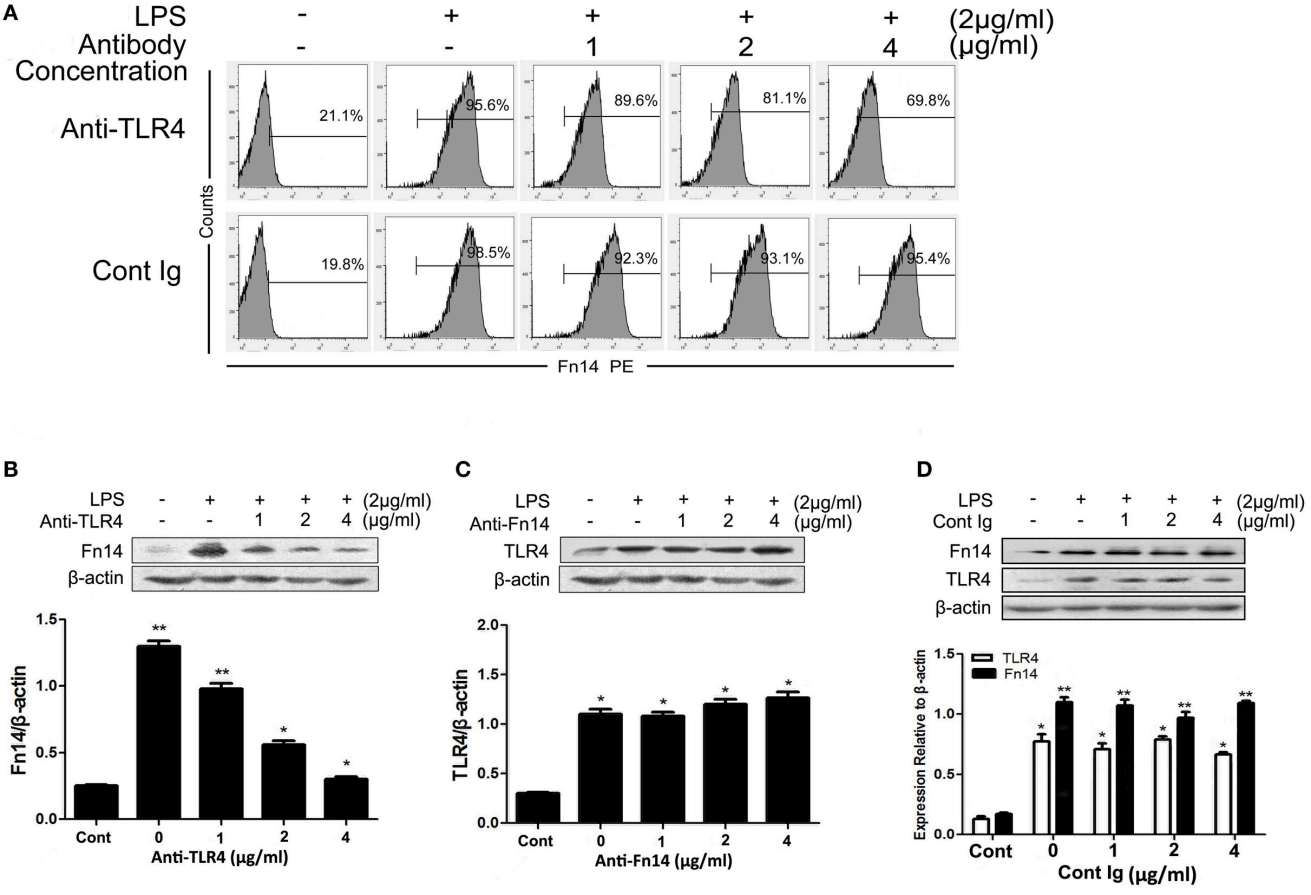
The expression of Fn14 and TLR4 protein in LPS-stimulated SIEC02 in the presence of anti-TLR4 or anti-Fn14 antibody. The porcine small intestine epithelial cell line SIEC02 were stimulated with LPS in the presence of increasing concentrations of either anti-TLR4 or anti-Fn14 antibody for 24 h, and flow cytometric assay was used to detect the super Fn14 expression **(A)** and Western blot assay was used to detect the total cellular expression levels of Fn14 **(B)** or TLR4 **(C)** protein in the cells. The expression of either Fn14 or TLR4 in SIEC02 treated with different concentrations of isotype antibody was used as control **(D)**. Representative blots are shown in the upper panel and the histograms in the under panel show the results of densitometric analysis of Fn14 and TLR4 expression (normalized to β-actin to correct for protein loading). The data represent the mean ± SEM (*n* = 3). ^*^P < 0.05, ^**^P < 0.01, compared with data on untreated cells.

In order to determine whether TLR4 regulates Fn14 expression or be regulated by this receptor, we next compared the expression levels of Fn14 and TLR4 in LPS-stimulated SIEC02 under different treatment conditions. As shown in Figures 3A,B, although LPS stimulation significantly increased the ratio of Fn14-positive cells as well as Fn14 levels detected by flow cytometry and Western blot analysis, respectively, neutralization of TLR4 activity with a specific antibody caused a significant down regulation of Fn14 expression in SIEC02 in a dose-dependent manner. In contrast, there are no any significant differences in TLR4 expression in LPS-stimulated SIEC02 treated with different concentrations of Fn14 blocking antibody (Figure 3C). Moreover, no significant difference in the expression of either Fn14 or TLR4 was detected in SIEC02 treated with different concentrations of isotype antibody control (Figure 3D).

**Figure 4:**
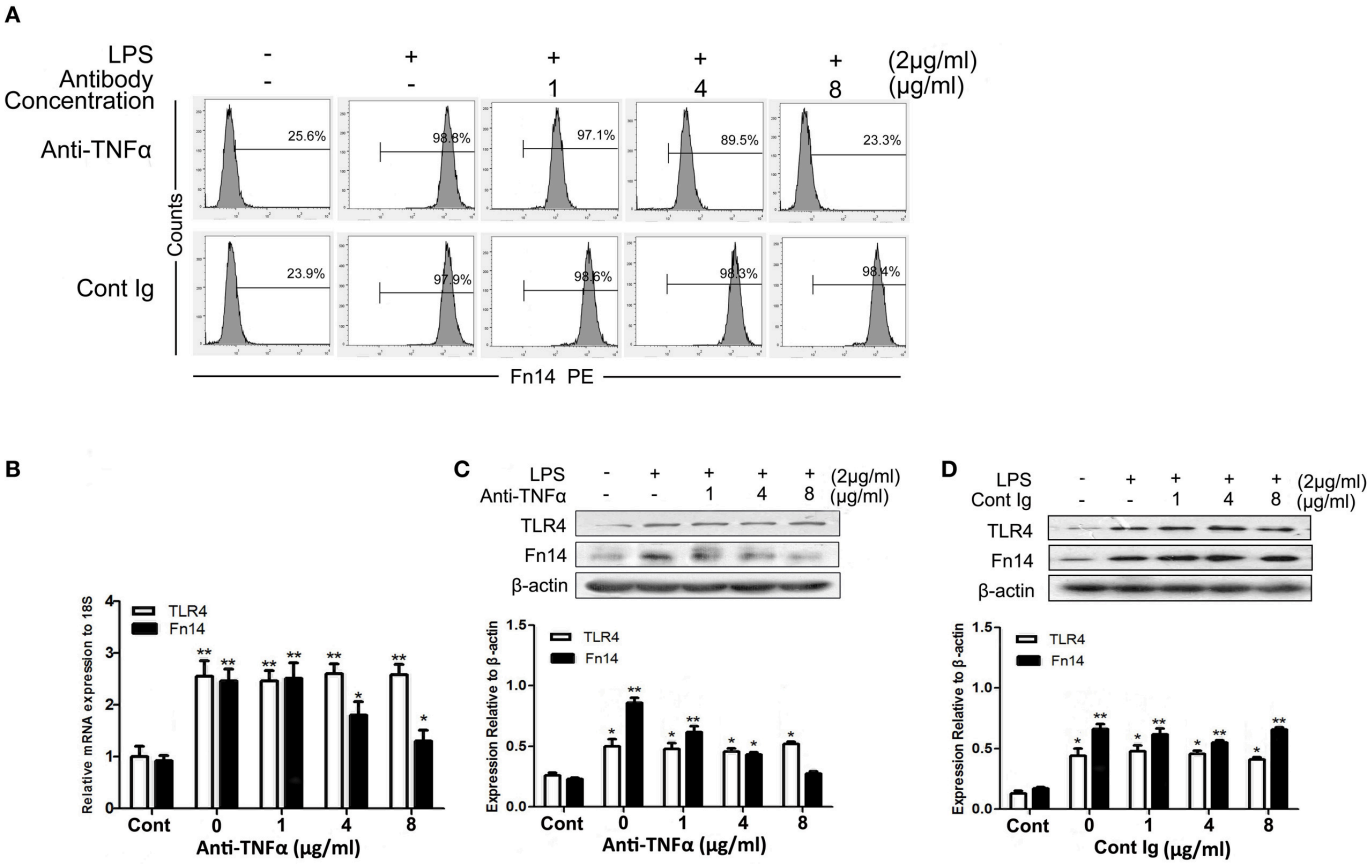
The expression of Fn14 and TLR4 protein in LPS-stimulated SIEC02 in the presence of anti-TNF-α antibody. The porcine small intestine epithelial cell line SIEC02 were stimulated with LPS in the presence of increasing concentrations of anti-TNF-α antibody for 24 h, and the super Fn14 expression on cells were analyzed by flow cytometric assay **(A)**. The expression levels of Fn14 and TLR4 were analyzed by quantitative real-time RT-PCR **(B)** and Western blot analysis **(C)**. The protein expression of either Fn14 or TLR4 in SIEC02 treated with different concentrations of isotype antibody was used as control **(D)**. Representative blots are shown in the upper panel and the histograms in the under panel show the results of densitometric analysis of Fn14 and TLR4 expression (normalized to β-actin to correct for protein loading). The data represent the mean ± SEM (*n* = 3). ^*^P < 0.05, ^**^P < 0.01, compared with data on untreated cells.

As the dose-dependent down regulation of Fn14 levels in LPS-stimulated SIEC02 in response to TLR4 blocking antibody treatment, we next determined whether activation of TLR4 signaling was responsible for the up regulation of Fn14 expression in LPS-stimulated SIEC02. The activation of TLR4 can mainly stimulate the production of TNF-α, then, we further examined the effects of neutralization of TNF-α on the Fn14 and TLR4 expression levels in LPS-stimulated SIEC02. Flow cytometric detection revealed that, antibody neutralization of TNF-α decreased the counts of Fn14-positive cells in LPS-induced SIEC02 in a dose-dependent manner (Figure 4A). Furthermore, treatment with a TNF-α antibody also decreased LPS-induced Fn14 expression levels in SIEC02 both at the mRNA and the protein levels in a dose-dependent manner (Figures 4B,C). However, neutralization of TNF-α has no significant effects on LPS-induced expression levels of TLR4 by SIEC02 (Figures 4B,C). There was no significant difference both in Fn14 and TLR4 protein expression by SIEC02 treated with different concentrations of isotype antibody control (Figure 4D).

### LPS-induced activating of ligand-independent Fn14 pathway

**Figure 5:**
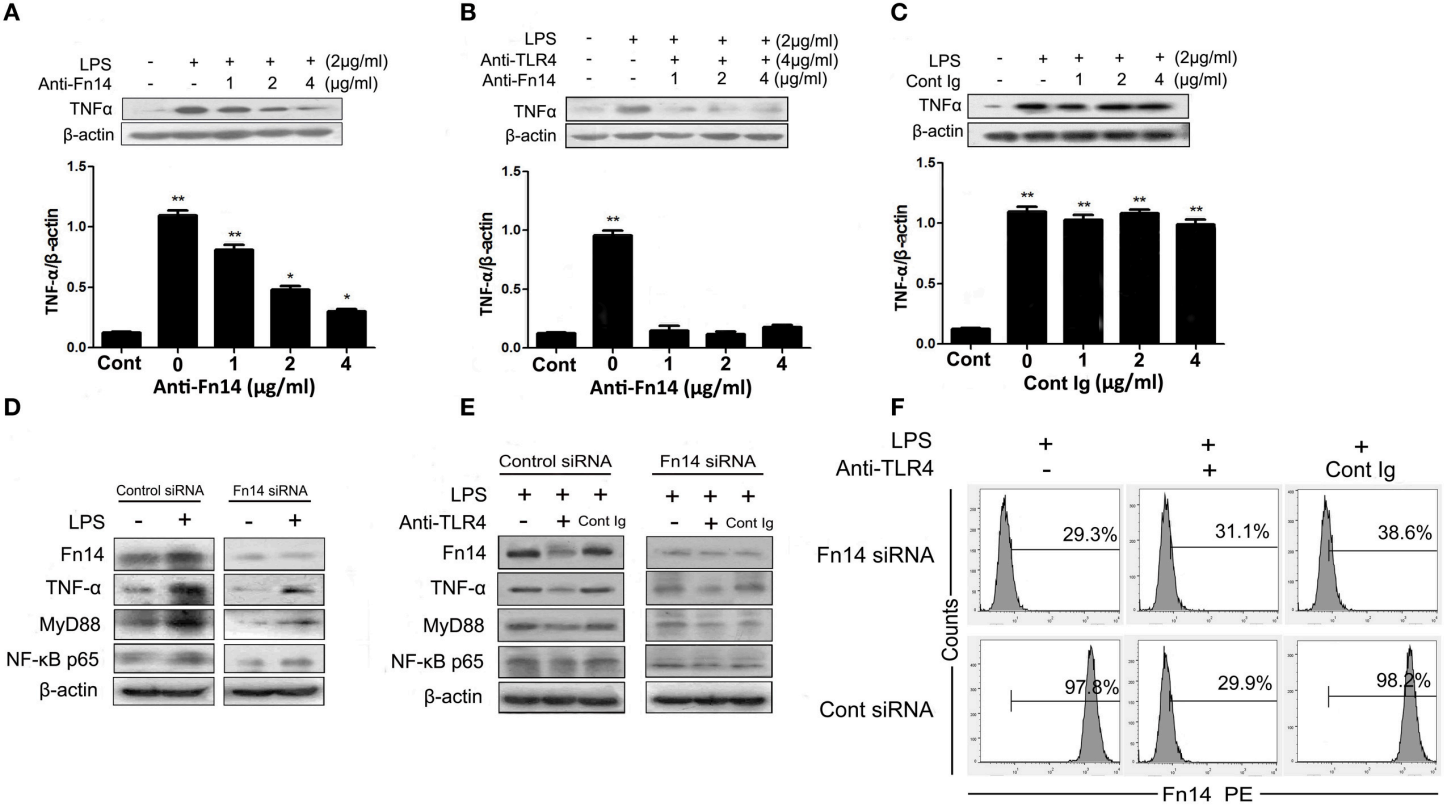
The cross-talk between the TLR4 and Fn14 modulate TNF-α expression in LPS-stimulated SIEC02. Western blot analysis of TNF-α expression in the LPS-stimulated SIEC02 in the presence of increasing concentration of anti-Fn14 **(A)** or both anti-Fn14 and anti-TLR4 **(B)**. The expression of TNF-α in SIEC02 treated with different concentrations of isotype antibody was used as control **(C)**. Western blot analysis of the activity of MyD88 and NF-κB pathways in siRNA-mediated Fn14 gene knockdown SIEC02 stimulated with LPS in the absence **(D)** or in the presence of **(E)** TLR4 antibody. Representative blots are shown in the upper panel and the histograms in the under panel show the results of densitometric analysis of indicated gene expression (normalized to β-actin to correct for protein loading). The data represent the mean ± SEM (*n* = 3). ^*^P < 0.05, ^**^P < 0.01, compared with data on untreated cells. Super Fn14 expression on siRNA-mediated Fn14 gene knockdown SIEC02 stimulated with LPS in the absence or in the presence of TLR4 antibody was analyzed by flow cytometric analysis **(F)**.

Similar to the results from *in vivo* studies, treatment of SIEC02 with LPS significantly increased the levels of Fn14, while there were no detectable levels of TWEAK both in LPS-stimulated and in untreated SIEC02 (data not shown). Next, we determined whether ligand-independent Fn14 signaling may amplified LPS-induced intestinal inflammation. To investigate this, SIEC02 were treated with the agonistic anti-Fn14 antibody, followed by stimulation with LPS. As presented in Figure 5A, stimulation with LPS significantly induced the expression of TNF-α in SIEC02 as assessed by Western blotting, whereas treatment with increasing concentrations of anti-Fn14 blocking antibody caused a dose-dependent decrease of TNF-α levels. However, in the presence of both anti-TLR4 antibody and anti-Fn14 antibody, no significant changes of TNF-α expression levels in LPS-stimulated SIEC02 were detected compared to the LPS untreated control cells (Figure 5B). This results suggests that Fn14 may augments LPS-induced inflammatory through TLR4 downstream signaling. The results obtained from isotype antibody control rule out the effects of Fc receptors which are expressed in intestinal epithelial cells (Figure 5C).

To explore if ligand-independent Fn14 activation in intestinal epithelial cells could activate MyD88-NF-κB pathways, we also examined the activity of MyD88 and NF-κB pathways in siRNA-mediated Fn14 knockdown SIEC02 stimulated with LPS. As presented in the Figure 5D, higher protein levels of TNF-α, MyD88, as well as canonical NF-κB component p65 (RelA) were detected in the LPS-stimulated control group (*P* < 0.05). However, Fn14 knockdown by siRNA resulted in a significant decrease of LPS-induced MyD88 and NF-κB p65, as well as TNF-α protein levels compared to that in control siRNA cells (Figure 5D). Moreover, although pretreatment of anti-TLR4 antibody decreased the expression levels of LPS-induced MyD88 and NF-κB p65, as well as TNF-α in control siRNA SIEC02 compared to the anti-TLR4 antibody untreated and isotype antibody control cells, low and no significant changes of expression levels were detected in Fn14 knockdown cells either stimulated with LPS or untreated (Figure 5E). Additionally, pretreatment with the agonistic anti-TLR4 antibody significantly decreased Fn14 expression levels in LPS-induced control siRNA SIEC02 as detected by Western blotting and flow cytometric analysis compared to antibody untreated and isotype antibody control cells (Figures 5E,F). However, Fn14 gene knockdown SIEC02 has low Fn14 expression levels and no significant difference between LPS-induced and untreated control cells (Figures 5E,F).

### TWEAK inhibits LPS-induced activating of Fn14 pathway

**Figure 6:**
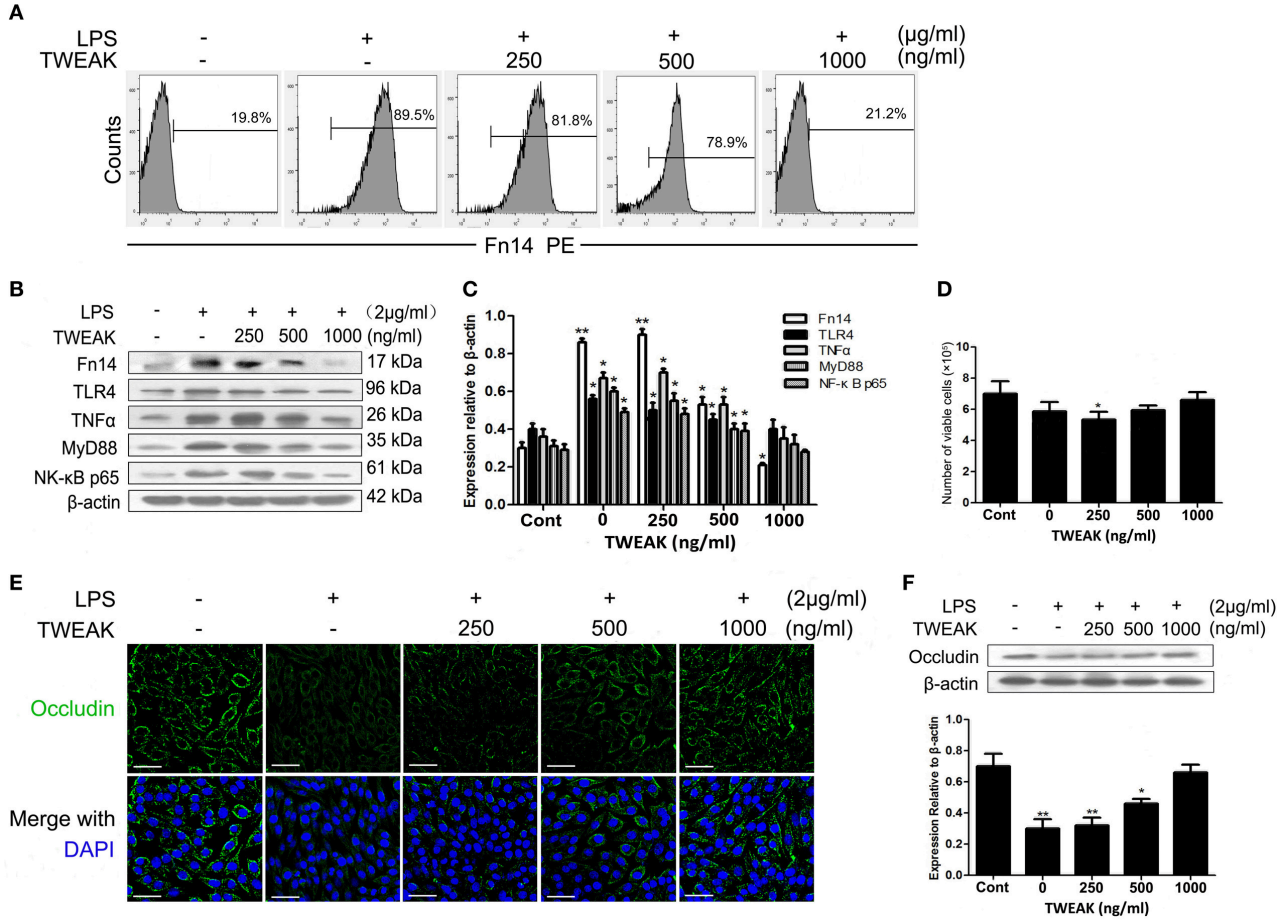
TWEAK inhibits the LPS-induced activating of Fn14 pathway. Treatment of porcine small intestinal epithelial cells (SIEC02) with recombinant TWEAK reduces LPS-induced super Fn14 expression **(A)** as well as TLR4/Fn14 expression by suppressing the activation of MyD88 and canonical NF-κB pathways in cells. Representative blots are shown in **(B)** and the histograms in **(C)** show the results of densitometric analysis of TNF-α expression in different treated SIEC02 (normalized to β-actin to correct for protein loading). SIEC02 seeded in a 24-well plate stimulated with LPS for 24 h were treated with recombinant TWEAK and after 24 h the number of viable cells was measured using the trypan blue exclusion method **(D)**. Representative fluorescence photomicrographs show localization of Occludin (green) in SIEC02 stimulated with LPS in the presence of increasing concentrations of recombinant TWEAK for 24 h **(E)**. Nuclei were counterstained blue with DAPI. Scale bars = 50 μm. The expression levels of Occludin were analyzed by Western blot analysis **(F)**. Representative blots are shown in the upper panel and the histograms in the under panel show the results of densitometric analysis of Occludin protein expression (normalized to β-actin to correct for protein loading). Data are expressed as mean ± SEM (*n* = 3). ^*^P < 0.05, ^**^P < 0.01, compared with data on untreated control cells.

As aforementioned results that treatment with LPS can down regulates TWEAK expression in intestinal tissues and that upregulation of Fn14 may augments LPS-induced inflammation in the intestine, we subsequently determined whether treatment of SIEC02 with recombinant TWEAK could suppress LPS-induced Fn14 expression in cells and reduce proinflammatory cytokines production. As indicated by the flow cytometry assay, continuous treatment with increasing concentrations of recombinant TWEAK for 24 h resulted in a dose-dependent decrease of surface Fn14 expression levels on LPS-induced SIEC02 (Figure 6A). Furthermore, similar to the expression pattern of Fn14, a does-dependent decrease of TNF-α and TLR4 level was also observed in LPS-stimulated SIEC02 in response to continuous treatment with increasing concentrations of TWEAK (Figures 6B,C). This results indicate that TWEAK may play a role in suppressing Fn14 mediated inflammation. Again, we examined the activity of MyD88 and NF-κB pathways in LPS stimulated SIEC02 in the presence or absence of TWEAK. As presented in the Figures 6B,C, compared with the untreated control cells, the LPS-treated SIEC02 displayed higher protein levels of MyD88 and NF-κB p65 (*P* < 0.05). Continuous treatment of SIEC02 with increasing concentrations of recombinant TWEAK resulted in a dose-dependent decrease of LPS-induced MyD88 and NF-κB p65 protein levels (Figures 6B,C).

To check whether the inhibitory effect of TWEAK on LPS-induced Fn14 as well as TIRAP-MyD88 signaling pathways relevant expression observed in the current study indeed reflects inhibition of LPS signaling and is not an epiphenomenon of TNF/TWEAK cytotoxicity, we determined the number of viable cells after 24 h TWEAK treatment by using trypan blue dye exclusion uptake assay. As shown in Figure 6D, collectively, there was no significant differences in the viability between recombinant TWEAK treated and untreated control cells.

### TWEAK blocks LPS-induced suppression of occludin expression in SIEC02

Given the previous findings that LPS regulates intestinal tight junction permeability through TLR4 pathway induced the activation of MyD88 (Guo et al., 2015), in combined with the present results that the upregulation of Fn14 may augments LPS-induced inflammation in the intestine, we further determined the changes of Occludin expression levels, a key intercellular tight junction protein in LPS-stimulated SIEC02 in the presence or absence of TWEAK treatment. As indicated by the immunofluorescence localization, we observed that Occludin staining appeared to be reduced and more fragmented around the cell border after stimulated with LPS, whereas the treatment of cells with TWEAK inhibited the reduction of Occludin staining in LPS-stimulated SIEC02 in a dose-dependent manner (Figure 6E). Furthermore, we used Western blot analysis to detect the expression of Occludin in SIEC02. Contrary to the expression pattern of MyD88, LPS stimulation significantly suppressed the expression of Occludin in SIEC02 as indicated by Western blot analysis. This LPS effect, however, was significantly inhibited in a dose-dependent manner by increasing concentrations of TWEAK treatment (Figure 6F).

## Discussion

Based on the use of multiple blocking antibodies and siRNA-mediated Fn14 knockdown experiments, our principal findings are that stimulation through TLR4 by its cognate ligand LPS, increases the expression of Fn14 *in vivo* and *in vitro*, and that activation of Fn14 in intestinal epithelial cells augments TLR4-mediated inflammation. These observations are significant, as they indicate an unappreciated role for Fn14 in intestinal immune responses.

It is well established that Fn14 is normally expressed at relatively low levels in healthy tissues, but Fn14 upregulation has been observed during tissue injury and inflammation (Burkly et al., 2011; Son et al., 2013). In particular, LPS stimulation induced the expression of Fn14 has been previously reported *in vitro* studies (Chacón et al., 2006; Qi et al., 2016). Similar results were observed in this study, we found that LPS-stimulated piglet have upregulated expression of TLR4 and Fn14 as well as decreased TWEAK expression in small intestine tissue compared to age matched untreated control piglets. It is expected that LPS stimulation resulted in the downregulation of TWEAK expression. The previous studies have indicated that the loss of TWEAK levels in responses to LPS treatment may attributed to the activation of a destabilizing factor acting on the AU-rich element of TWEAK mRNA (Chicheportiche et al., 2000). In addition, significant immunostaining of Fn14 was visibly stronger in the luminal epithelium of LPS-stimulated small intestine, which suggesting that TWEAK/Fn14 signaling pathway may occured mainly in the epithelium of intestine in response to the stimulation of LPS. One interesting aspect of Fn14 biology is that the highly inducible pattern of Fn14 expression at both *in vitro* and *in vivo* is detected. Although persistent upregulation of Fn14 in states of chronic inflammatory disease has been previously reported in rodent model (Son et al., 2013), to the best of our knowledge no report has demonstrated an upregulation of Fn14 by pathogen-activated molecular patterns TLR4 in intestinal epithelial cells. In this study, we concluded that LPS-induced TLR4 expression upregulated Fn14 expression in the small intestinal epithelial cells of piglets, whereas Fn14 had no significant effects on TLR4 expression. Given that Fn14 upregulation was shown to be induced by a variety of Fn14-inducing stimuli, including growth factors, cytokines, hormones and compounds (Winkles, 2008; Dohi et al., 2009; Kawashima et al., 2011), we speculate that TNF-α may, at least, partially mediate TLR4 regulation of Fn14 expression in SIEC02. As predicted, antibody neutralization of TNF-α decreased LPS-induced Fn14 expression levels in a dose-dependent manner, while has no significant effects on TLR4 expression. This study also demonstrate extensive cross-talk between the Fn14 and TLR4 signaling pathways in terms of TNF-α expression in response to LPS stimulation. Our data indicate that LPS-activated intestinal epithelial cells produce a large amount of TNF-α upon both TLR4 and Fn14 stimulation, while TNF-α levels were reduced in a dose-dependent manner in SIEC02 treated with increasing concentrations of anti-Fn14 antibody. Furthermore, Fn14 knockdown almost abrogate the production of TNF-α in LPS-induced SIEC02. This result implicated that LPS-induced TLR4 activation upregulates Fn14 expression and the activation of Fn14 may augment LPS-induced inflammation through TLR4 downstream signaling. The positive feedback loop that leads to amplified receptor expression by TNF-α results in a system that is very sensitive to TWEAK and, potentially also able to signal in a ligand-independent manner (Winkles, 2008; Brown et al., 2013).

TLR4 activates downstream signaling pathways in response to LPS stimulation mainly by two major pathway, including TIRAP-MyD88 and TRIF-TRAM pathway (Vaure and Liu, 2014). Interestingly, TIRAP-MyD88 signaling pathway induced the activation of NF-κB signaling has been shown to closely associated with the abnormal Fn14 expression (Winkles, 2008). We subsequently determined that in SIEC02, Fn14 activation may modulate MyD88-NF-κB pathways. We found that LPS stimulation upregulated Fn14 expression in SIEC02, which was accompanied by the increase of NF-κB p65 and MyD88 protein expression. However, Fn14 knockdown by siRNA resulted in a significant decrease of LPS-induced MyD88 and NF-κB p65, as well as TNF-α protein levels. Interestingly, pretreatment of anti-TLR4 antibody decreased the expression levels of LPS-induced MyD88 and NF-κB p65, as well as TNF-α both in Fn14 knockdown and control SIEC02 compared to the anti-TLR4 antibody untreated and isotype antibody control cells. Thus, the augmentation of TLR4-induced TNF-α expression seen upon Fn14 activation may be, at least in part, due to the further activation of MyD88-NF-κB pathways. In addition, we cannot rule out the possible effects of TRIF-TRAM pathway involved in Fn14 regulation. Further studies are needed to address this issue. It is interesting to note that, in our *in vitro* experimental system, TWEAK-dependent signaling is not mediating the observed cellular responses as no detectable TWEAK levels were detected. In consideration of this finding, as well as previous results demonstrating that other TNF receptor superfamily members can signal by their own when over expressed in cells (Xu and Shu, 2002; Fotin-Mleczek et al., 2004; Hauer et al., 2005), this study strongly suggests that when cellular Fn14 levels reach a certain threshold level, ligand-independent Fn14 signaling may be predominant.

Although a variety of factor can induce Fn14 expression, little is known about the factor that may determine the steady-state stability or downregulation of Fn14. Recently, Gurunathan et al. (2014) indicated that TWEAK treatment can downregulated Fn14 expression levels in HeLa cell lines. In the current study, we found that Fn14 undergoes ligand-dependent receptor downregulation in LPS-stimulated porcine small intestinal epithelial cells. In fact, it was previously demonstrated that Fn14 expressed on the cell surface undergoes rapid constitutive synthesis and degradation, however the presence of TWEAK could accelerate this degradation (Gurunathan et al., 2014). Given our findings that high TWEAK and low Fn14 expression in healthy small intestine tissues of piglets, we anticipate that TWEAK-dependent suppression of Fn14 signaling may play a role in intestinal homeostasis, as signaling from the commensal bacteria in intestinal epithelia via TLR4 regulates Fn14 expression (Fernandes et al., 2014). However, if the expression of TWEAK is downregulated while Fn14 elevated in a variety of acute and chronic inflammatory pathologies (Chicheportiche et al., 2000), then TWEAK-independent Fn14 signaling can occur. Furthermore, it has been shown that TNF and TWEAK synergistically induce cell death in intestinal epithelial cells (Chopra et al., 2015). Since LPS induces TNF in the intestinal cells investigated here, cell death could play a role in LPS/TWEAK co-treated cells. Our study demonstrated that there was no significant difference in the viability between recombinant TWEAK treated and untreated control cells. One possible explanation is that the synergistical effect of TNF and TWEAK on cell death in intestinal epithelial cells may simply vary dependending on the doses or the experimental systems to which it is added.

It was previously suggested that the physiological concentrations of LPS cause an increase in intestinal TJP via a TLR4 signal transduction pathway-dependent process (Guo et al., 2013). Of interest, a recent *in vivo* study confirmed that high-dose LPS-stimulated increase of TJP in intestine as well as intestinal inflammation were via the TLR4/MyD88 signal transduction axis (Guo et al., 2015). Based on our findings that upregulation of Fn14 augment LPS-induced inflammation and downregulate Occludin expression in SIEC02 via activation of MyD88-NF-κB pathways, we extend previous findings to demonstrate that Fn14 may play a role in augmenting LPS-induced TJP. Additionally, our study showed that TWEAK pretreatment inhibited the negative impact of LPS on the tight junctional protein Occludin expression in SIEC02, which indicates that TWEAK may play a role in modulating Occludin expression in LPS-stimulated SIEC02.

In summary, we have shown that extensive cross-talk exists between Fn14 and TLR4 in LPS-stimulated intestinal epithelial cells *in vitro*. Our data further advance the evidence of augmentation of inflammation of Fn14 and reveal a link between ligand-independent Fn14 signal and the presence of LPS in porcine small intestinal epithelial cells. The alterations in TLR4-mediated TNF-α production shown in this study, upon manipulation of both the expression and the activation of Fn14, suggest that targeting Fn14 has potential therapeutic applications and warrants further investigation.

## Author contributions

XQ and LQ performed the majority of experiments. RD, YC, MD, and ML participated part of the experiments. XQ conceived the study, participated in its design and coordination. XQ and JW prepared and revised the manuscript. All authors have read and approved the final manuscript.

### Conflict of interest statement

The authors declare that the research was conducted in the absence of any commercial or financial relationships that could be construed as a potential conflict of interest.
